# Chaotic Motifs in Gene Regulatory Networks

**DOI:** 10.1371/journal.pone.0039355

**Published:** 2012-07-06

**Authors:** Zhaoyang Zhang, Weiming Ye, Yu Qian, Zhigang Zheng, Xuhui Huang, Gang Hu

**Affiliations:** 1 Department of Physics, Beijing Normal University, Beijing, China; 2 School of Management, Beijing Normal University, Beijing, China; 3 Nonlinear Research Institute, Baoji University of Arts and Sciences, Baoji, China; Humboldt University, Germany

## Abstract

Chaos should occur often in gene regulatory networks (GRNs) which have been widely described by nonlinear coupled ordinary differential equations, if their dimensions are no less than 3. It is therefore puzzling that chaos has never been reported in GRNs in nature and is also extremely rare in models of GRNs. On the other hand, the topic of motifs has attracted great attention in studying biological networks, and network motifs are suggested to be elementary building blocks that carry out some key functions in the network. In this paper, chaotic motifs (subnetworks with chaos) in GRNs are systematically investigated. The conclusion is that: (i) chaos can only appear through competitions between different oscillatory modes with rivaling intensities. Conditions required for chaotic GRNs are found to be very strict, which make chaotic GRNs extremely rare. (ii) Chaotic motifs are explored as the simplest few-node structures capable of producing chaos, and serve as the intrinsic source of chaos of random few-node GRNs. Several optimal motifs causing chaos with atypically high probability are figured out. (iii) Moreover, we discovered that a number of special oscillators can never produce chaos. These structures bring some advantages on rhythmic functions and may help us understand the robustness of diverse biological rhythms. (iv) The methods of dominant phase-advanced driving (DPAD) and DPAD time fraction are proposed to quantitatively identify chaotic motifs and to explain the origin of chaotic behaviors in GRNs.

## Introduction

In systems biology, gene regulatory networks (GRNs), a kind of biochemical regulatory networks, are widely described by coupled differential equations (ODEs) [1]–[9]. These ODEs are strongly nonlinear and often high-dimensional. Meanwhile, gene regulatory networks also share some common characteristics and network structures. For instance, positive feedback loops (PFLs) and negative feedback loops (NFLs) have been identified in various biochemical regulatory networks and found to be important control modes in GRNs. It has been demonstrated that NFLs can act as noise suppressors [6]–[8], oscillators [4], [6]–[9], and linearizers [8], and PFLs can behave as switches and memory modules [7], [10]–[13]. Since multiple and coupled oscillators are likely to be common companions in the intricate GRNs [4], complex nonlinear dynamic behaviors, such as self-sustained oscillation, birhythmicity, bursting oscillation and even chaos, are reasonably expected for these objects [2], [14]–[19].

According to our understanding on the theory of nonlinear dynamical systems, strongly coupled high dimensional ODEs are very likely to show chaotic behaviors. Especially, chaos has been found in some chemical reactions both in experiments and simulations, such as the Peroxidase-Oxidase reaction [20]–[23] and the Belousov-Zhabotinsky reaction [23]–[26]. Therefore, chaos may occur often in 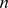-dimensional (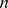
D) GRNs with 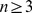. However, chaos is extremely rare in GRNs and have seldom reported with 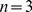 [18], [27]. These results are surely beneficial from biological viewpoint, however, the reason for this rareness has still not been fully understood [15]–[17], [19].

Recently, the topic of motifs has attracted great interest in studying biological networks [28]–[32]. Network motifs are subgraphs appearing in some biological networks far more often than in randomized networks and they are suggested to be elementary building blocks that carry out some key functions in the network. Various types of motifs producing some simple functions have been explored and studied, such as sign-sensitive accelerators or delays of feed-forward loop [30], tunable biological oscillations of coupled NFL and PFL [13], and so on.

Dynamical motifs (subnetworks with nontrivial dynamics) have been presented as a new approach to the study of the dynamics in networks [33]. We apply the concept of motifs to investigate the chaotic dynamics in GRNs. Chaotic motifs are those minimal structures with the simplest interactions that can generate chaos. It has been demonstrated that complex oscillatory behavior, such as birhythmicity, bursting and chaos, is due to the competition between two oscillatory mechanism with a comparable importance [2], [4], [17]. Chaos has also been found in a simple three-variable biochemical system, which is only comprised of two feedback loops [18]. However, it is still unclear that, in what degree network structures can determine the existence of chaotic behaviors in GRNs. In other words, there is no general conclusion on the relationship between network structures and chaotic dynamics so far.

In this paper, we concentrate on chaotic behaviors of autonomous GRNs and address the above issues by answering the following questions: considering the crucial conditions for chaos in GRNs, why chaos is so rare; and are there some simple patterns namely chaotic motifs in complex GRNs serve as the basic building blocks for chaotic motions. We extensively search for chaos in low-dimensional GRNs (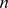
D with 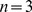, 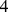 and 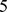) and study the mechanism of chaos. The key results of our study are: we do find various chaotic motifs which behave as the minimal building blocks or subnetworks related to chaotic motions, where competitions between different oscillatory modes serve as the necessary condition for chaos. Based on the competition idea, we reveal the conditions under which chaos can be easily found, even we can readily explore some chaotic 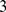
D GRNs where chaos has never been reported in the previous investigations. Furthermore, a number of special structures are discovered which can never generate chaos, and these structures may be related to different biological oscillators which bring some advantages on rhythmic functions avoiding chaotic motions definitely. Finally, we apply the methods of dominant phase-advanced driving (DPAD) [34], [35] and DPAD time fraction to give some quantitative measurements explaining the above results on chaotic GRNs.

## Results

### Rareness of Chaos

We search chaotic motions extensively in low dimensional GRNs described by ODEs (refer to Materials and Methods for the detail of the model and searching). Each case (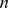
D with 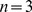, 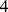 and 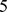, respectively) has 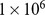 samples from random network structures, parameter distributions and initial variable conditions. The asymptotic states are finally recorded. The states are classified into three different types: steady states, periodic oscillations and chaotic motions. The results are presented in Table 1. It is observed that while most of the tests approach to steady states, much less (but still many) tend to periodic oscillations. The asymptotic chaotic states are extremely rare indeed.

**Table 1:** Attractor distributions in random GRNs.

Random	3-node	4-node	5-node
**Oscillation**	1.233%	1.775%	2.432%
**Chaos**	1	20	42

Asymptotic states reached by 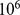 tests with random network structures, parameter distributions and initial variable conditions for 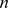
D GRNs with 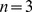, 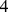 and 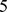. The asymptotic states are classified into three types: steady, periodic oscillatory and chaotic states. Most tests tend to stable steady states and much less periodic oscillations. The probability of oscillations is computed out, and the amount of chaotic samples is counted out of every 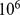 tests. The asymptotic chaotic states are extremely rare indeed.

Now, our next task is to enlarge the chaotic samples for detailed investigation of chaotic GRNs. The following strategies are applied: (i) Some period-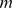 (called 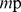) states with 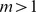 are discovered in the above periodic oscillations. With the clues of all these 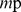 states, chaotic solutions can be easily obtained by continually varying parameters (various bifurcation sequences to chaos). Therefore, all the 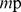 states with 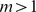 will be simply classified as chaotic solutions in the following investigations. (ii) Since the existence of oscillation is a necessary condition for chaos, much more effective searching can be made among all these periodic GRNs. In Table 2, we search for chaos in the way exactly the same as in Table 1 by randomly choosing parameters and initial conditions but within the classes of periodic networks in Table 1. In Table 2, considerably richer chaotic behaviors are observed than that of Table 1, and all these samples can be used for studying the mechanism of chaos in GRNs.

**Table 2:** Attractor distributions in oscillatory networks in Table 1.

Periodic	3-node	4-node	5-node
**Oscillation**	41.157%	40.158%	39.310%
**Chaos**	195	1158	1591

The same as Table 1 with all data measured within the network structures of periodic oscillations in Table 1. Since the existence of oscillation is a necessary condition for chaos, much more effective searching can be made among all the periodic GRNs. Obviously, we do observe considerably richer chaotic behaviors than that of Table 1. The amount of chaotic samples is also counted out of every 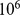 tests, and all these chaotic samples will be used in the following for studying the mechanism of chaos.

It can be inferred from Table 2 that oscillations in our model are rather robust against parameter sets. More than forty percent of the samples from periodic network structures in Table 1 remain in oscillatory states by randomly varying parameters and initial variable conditions.

### Introduction to Chaotic Motifs

**Figure 1:**
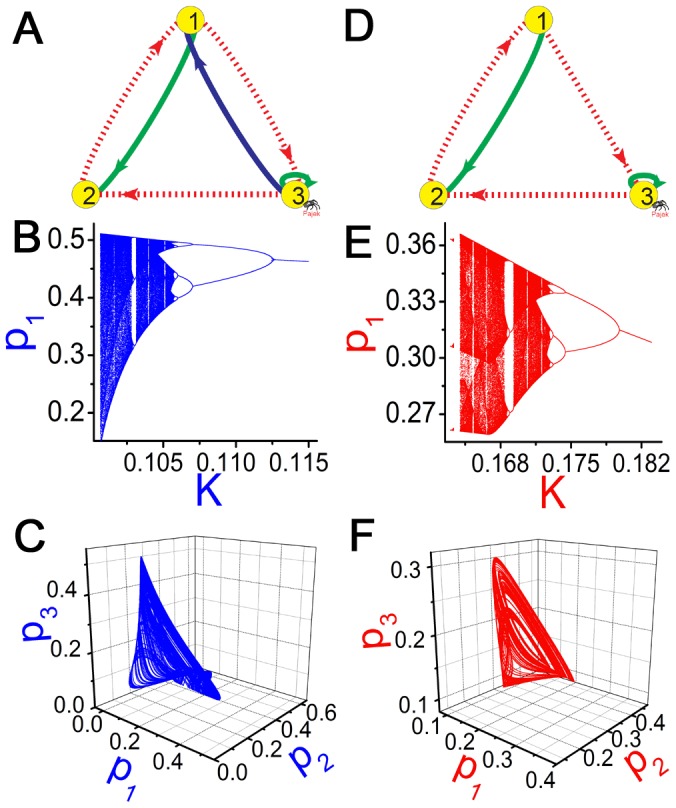
A 3-node chaotic motif. A, D: Two 3-node chaotic GRNs. Solid (dashed) lines denote active (repressive) regulations. B, E: Bifurcation diagrams of A and D, respectively (peak values of 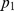 plotted as functions of 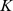 with 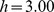). C, F: Chaotic attractors (C for A with 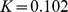, F for D with 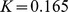). From A to D, we discard a single interaction (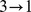, blue line in A). The removal does not essentially affect the chaotic motion. The bifurcation diagrams and the chaotic attractors of the two GRNs in A and D are similar. On the other hand, all the cross interactions in D (cross refers to interaction between different nodes) are irreducible, and removal of any of them can surely suppress chaos no matter how to adjust the parameters, initial conditions and self-regulations of different nodes. The cross interaction structure of D is identified as a 3-node chaotic motif.

We analyze the chaotic behaviors of some chaotic samples in Table 2 to study the mechanism of chaos. A 3-node GRN is plotted in Fig.1A which can exhibit chaos by varying parameters shown in Fig.1B. A chaotic attractor of this GRN is presented in Fig.1C. Let us consider a biologically relevant problem which interactions in Fig.1A are crucial for chaos, or in other words, what is the minimal structure of Fig.1A to produce chaos. In order to do this, different interactions are deleted in different tests and the structure of Fig.1D is found finally by deleting a single interaction 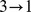 from Fig.1A (solid (dashed) arrows denote active (repressive) interactions), in which chaos can be still maintained. Further deleting any cross coupling (cross refers to interaction between different nodes) from Fig.1D can definitely suppress chaos no matter how to vary system parameters, initial conditions and self-regulations. It is interesting that the two GRNs in Figs.1A and 1D show similar types of bifurcation sequences to chaos (Figs.1B and 1E) and persist alike chaotic attractors (Figs.1C and 1F). These observations illustrate that the deleted interaction from Fig.1A to Fig.1D is not essential for the chaotic behaviors of Figs.1B and 1C. Conversely, the remaining cross interactions (the two feedback loops) in Fig.1D are all crucial for chaotic motion. Therefore, we consider all the minimal structures of cross interactions similar to Fig.1D as 3-node chaotic motifs of GRNs, in which removing any single cross interaction can surely destroy chaos.

**Figure 2:**
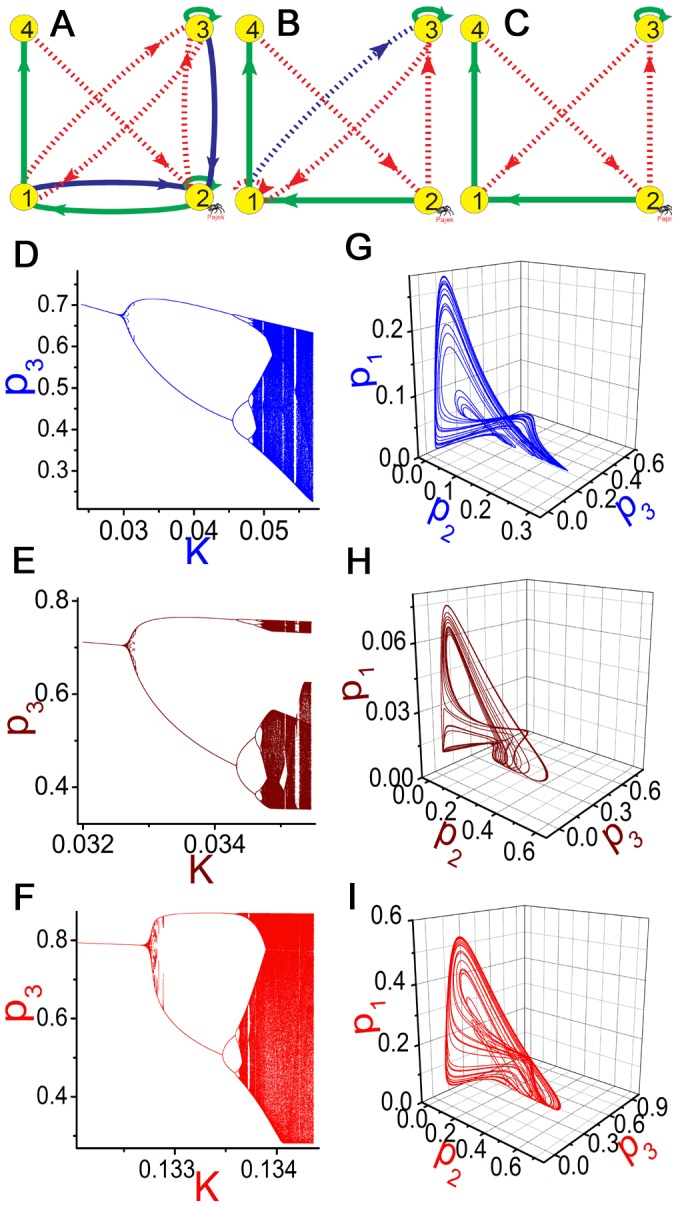
A 4-node chaotic motif. The same as Fig.1 with three 4-node GRNs considered. A-C: The GRNs under investigations. D−F: Their corresponding bifurcation diagrams (D for A with 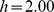; E for B with 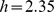; F for C with 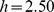). G-I: The corresponding chaotic attractors (G for A with 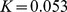; H for B with 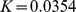; I for C with 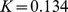). From A to B the interactions 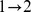 and 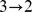 are removed, and from B to C the interaction 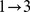 is deleted. These deletions do not essentially affect the chaotic motions. All bifurcation diagrams to chaos (D−F) and chaotic attractors of the three GRNs (G−I) are similar. Network C is irreducible for chaos in the sense that removal of any cross interaction of it can absolutely terminate chaos. The cross interaction structure of C is thus considered as a 4-node chaotic motif.

Similar concept can be also defined for 4-node GRNs. In Fig.2, we do exactly the same as Fig.1 with three 4-node GRNs (Figs.2A-2C) considered. From Fig.2A to Fig.2B and from Fig.2B to Fig.2C, we delete two interactions 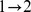 and 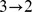 and another interaction 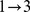 (blue lines in the GRNs). All the three GRNs display similar bifurcation sequences to chaos (Figs.2D−2F) and persist alike chaotic attractors (Figs.2G−2I). These observations demonstrate again that the deleted interactions are not essential for chaos. It is found further that all the cross interactions in Fig.2C are crucial for chaos, and removing any of them can entirely suppress chaos. We consider the cross interacted structure of Fig.2C and all similar minimal 4-node subnetworks as 4-node chaotic motifs.

### Conditions for Chaotic Motifs

It is of special importance to investigate what kinds of GRNs can serve as chaotic motifs. We define chaotic motifs as those irreducible chaotic subnetworks which have the fewest feedback loops and the least cross interactions (such as GRNs of Figs.1D and 2C). In each chaotic motif, the cross interactions are all crucial for chaotic behaviors, in another words, chaos would be lost by removing any of the cross interactions, no matter how to vary system parameters, initial conditions and self-regulations. Therefore, a basic motif is just defined as the structure of the cross interactions.

Some necessary conditions for chaotic motifs can be reasonably expected. The first crucial requirement of chaos is the competition between two or more oscillatory modes [2], [4], [19]. It is more or less known that feedback loops may represent different oscillatory modes in GRNs [6], [8], [9], [12], [18], [36]. Therefore, the existence of at least two feedback loops in GRNs, generating different oscillatory modes in competition, must be fulfilled by chaotic GRNs. Moreover, self-sustained oscillation is also a basic condition for chaos. Since the absolutely necessary condition for self-sustained oscillation of GRNs is existence of, at least, a NFL [4], [7], [9], and this is the second condition for chaos.

Therefore, all the chaotic motifs may possess the following two characteristic features: (i) All these motifs have two feedback loops (the fewest feedback loops for chaotic motifs); (ii) At least one of the two loops is NFL. We exhaustively search all 3-node and 4-node GRNs with minimal (irreducible) interactions satisfying the above two conditions, and eventually find 19 distinct 3-node and 86 distinct 4-node candidates of chaotic motifs. Fig. S1 shows a complete list of all these 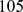 two-loop structures (TLSs).

**Figure 3:**
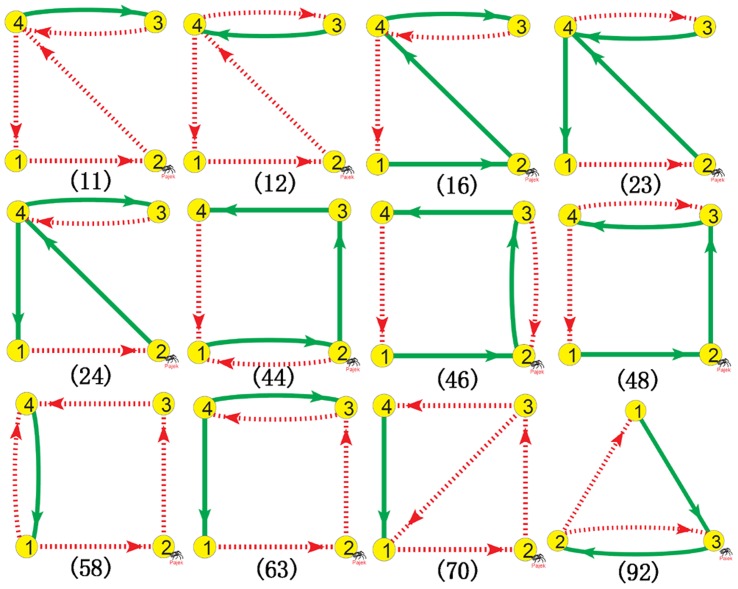
Significant chaotic motifs. By chaotic motifs, we mean that these GRNs can produce chaos with certain parameters, initial conditions and suitable self-regulations, and removal of any cross interaction in these GRNs can definitely terminate chaos. Chaotic motifs are the minimal and irreducible building blocks for chaotic motions in GRNs. Some significant motifs are shown with amount of chaotic realizations more than 100 in Table 3. There are four conditions (i)−(iv) for chaotic motifs. All these motifs possess two feedback loops (i), and at least one of them is NFL (ii). In all the motifs which contain one PFL and one NFL, the PFL must have at least one node not included in the NFL (iii). In most of the chaotic motifs, there is a node regulated by both repressive and active regulations (iv). If not, the motifs more often contain two NFLs.

With all the 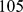 TLSs, we can do the same as in Table 1 to search for chaos. A number of chaotic motifs are discovered after 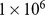 tests for each TLS by varying system parameters, initial conditions and self-regulations of the structure randomly. Asymptotic state distributions of the chaotic motifs in the random tests are shown in Table 3. Besides, some significant chaotic motifs are shown in Fig.3 which show, among 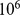 random tests, more than 100 chaotic realizations in Table 3.

**Table 3:** Attractor distributions in chaotic motifs.

Motif	(1)	(4)	(7)	(8)	(9)	(11)	(12)	(15)
**Oscillation**	0.119%	<0.10%	<0.10%	0.127%	3.891%	1.963%	4.062%	5.375%
**Chaos**	16	1	1	2	10	373	103	7
**Motif**	**(16)**	**(17)**	**(19)**	**(20)**	**(21)**	**(23)**	**(24)**	**(25)**
**Oscillation**	4.414%	4.512%	8.105%	4.915%	2.634%	4.370%	5.366%	0.158%
**Chaos**	1145	20	12	11	1	469	252	16
**Motif**	**(26)**	**(28)**	**(29)**	**(30)**	**(32)**	**(34)**	**(35)**	**(39)**
**Oscillation**	8.091%	1.926%	<0.10%	<0.10%	0.153%	4.735%	3.254%	3.844%
**Chaos**	15	12	1	1	4	73	49	65
**Motif**	**(40)**	**(41)**	**(44)**	**(46)**	**(48)**	**(52)**	**(54)**	**(56)**
**Oscillation**	3.242%	3.848%	5.006%	5.703%	4.552%	9.295%	10.164%	11.016%
**Chaos**	1	54	158	198	928	1	1	32
**Motif**	**(58)**	**(59)**	**(61)**	**(63)**	**(65)**	**(67)**	**(70)**	**(72)**
**Oscillation**	2.204%	1.469%	4.570%	3.634%	9.760%	10.321%	6.316%	5.283%
**Chaos**	111	41	12	283	3	19	123	1
**Motif**	**(73)**	**(74)**	**(77)**	**(78)**	**(79)**	**(80)**	**(82)**	**(83)**
**Oscillation**	5.730%	2.137%	10.803%	6.704%	1.321%	2.530%	10.925%	12.067%
**Chaos**	15	5	33	2	5	9	1	29
**Motif**	**(87)**	**(92)**	**(95)**	**(100)**	**(103)**	**(104)**	**(105)**	
**Oscillation**	5.487%	5.825%	<0.10%	0.448%	<0.10%	<0.10%	0.158%	
**Chaos**	16	182	15	22	1	1	6	

Classifications of asymptotic states reached by 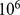 tests for each of the 105 TLSs in Fig. S1 with random parameter distributions, initial variable conditions and self-regulations. TFLs which can produce chaos (chaotic motifs) in the above tests are listed in the Table. More motifs can be found with more extensive searching among the rest of TFLs, however, their probability is extremely low. The amount of chaos is counted out of every 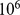 tests. The indexes of the motifs are given in Fig. S1.

With further investigation on the network structures of the chaotic motifs in Fig.3 and Table 3, two additional characteristic features for chaotic motifs are discovered: (iii) If a motif contains one NFL and one PFL, the PFL must contain at least one node not included in the NFL. (iv) In most of the chaotic motifs in Fig.3 and Table 3, there is one node which is regulated by joint cross interactions of both active and repressive couplings.

Extensive numerical simulations with these 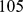 TLSs show that items (i)−(iii) are necessary conditions for chaotic motifs (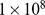 random tests have been made, none of these chaotic samples violates any of the three conditions). Condition (iv) is not the necessary condition for chaotic motifs. Specifically, we can distinguish all the TLSs which satisfy all conditions (i)−(iii) into three types, type 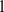: satisfy condition (iv) with a node regulated jointly by an active and repressive interactions; type 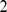: violate condition (iv), with two NFLs; type 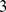: violate condition (iv), with one NFL and one PFL. Within all the chaotic subnetworks discovered in Table 3, we found that type 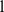 has 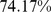 probability; type 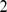, 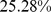; while type 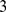, 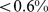. We found numerically that conditions (i)−(iv) comprise the sufficient conditions for 3-node and 4-node chaotic motifs, in other words, all the TLSs which satisfy the four conditions (i)−(iv) can generate chaotic motions with suitable self-regulations, parameter sets and initial variable conditions.

The reason for condition (iii) can be intuitively understood based on the the competition between different oscillatory modes. It is known that NFLs can support self-sustained oscillations while PFLs alone can not [3], [4], [9]. If there are one PFL and one NFL in a TLS and all the nodes of the PFL are included in the NFL, the PFL is completely controlled by the NFL. Therefore, the PFL can not essentially influence the oscillation of the NFL, and the TLS can not yield effective oscillatory competition to generate chaos.

**Figure 4:**
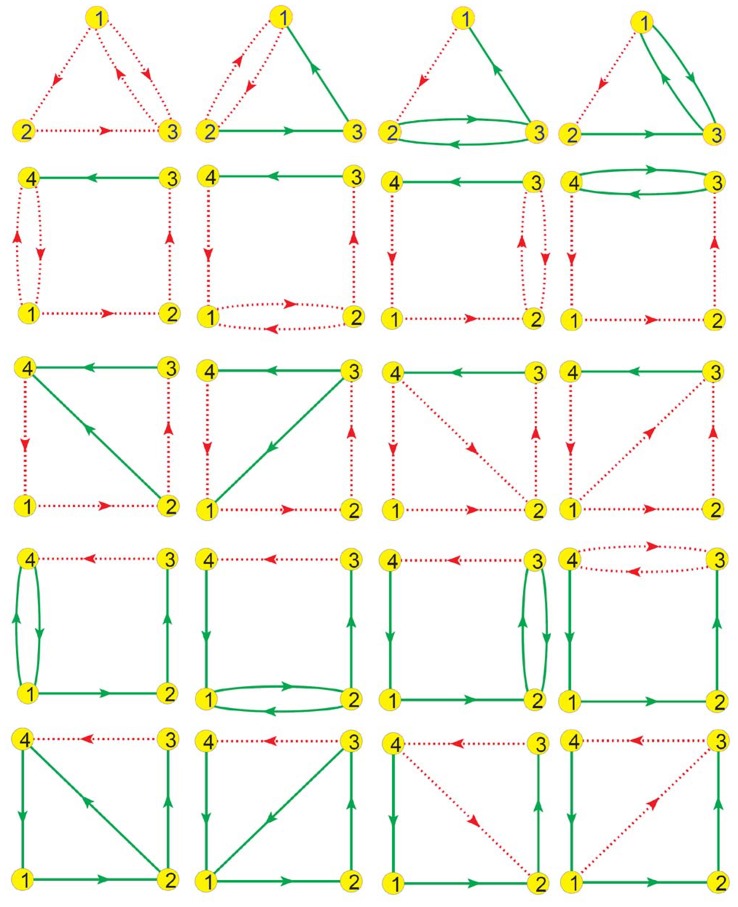
Regular rhythmic TLSs. All 3-node and 4-node TLSs, which fulfill conditions (i) and (ii) while violate condition (iii), can never produce chaos. The competition between the two feedback loops in each GRN does not work for chaos due to the fact that the PFLs are completely controlled by the corresponding NFLs. Therefore, all these TLSs can never produce chaos. For each of these TLSs, more than 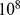 random tests are made by varying self-regulations, parameter sets and initial conditions, and none of these tests yields chaos. These structures may bring some advantages on rhythmic functions, avoiding the chaotic disturbances definitely.

All 3-node and 4-node TLSs which satisfy conditions (i) and (ii) while violate condition (iii) are listed in Fig.4. For these 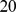 TLSs, above 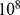 random tests are made for each one by changing self-regulations, initial conditions and all the parameters. Chaos is not observed among any of them. The regular rhythmic subnetworks of Fig.4 may permit some advantages on various important biological functions. Some of these structures may appear in certain biological processes to keep the rhythmicity regular, and it is worthwhile introducing to biological experimental scientists the structures of Fig.4 in which chaos is entirely avoided.

The mechanism underlying condition (iv) is not yet clear. Competition between the two loops of chaotic motifs is essentially determined by the dynamics of the dual-input nodes (e.g., node 2 in Fig.1D, node 1 in Fig.2C, and so on). An interesting observation is that in all the 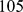 TLSs, each structure has only a single node regulated by two neighbor nodes, called as center node (all the other nodes in the structure receive only single inputs from cross interactions). We guess that the active and repressive regulations of center nodes may be favorable to strengthen the competition between the two loops, especially when the chaotic motifs contain one PFL and one NFL. It is emphasized that TLSs with two NFLs may generate two self-sustainedly oscillatory modes which can more strongly compete to produce chaos, even if the double inputs of center nodes have same signs (motifs (11), (23) in Fig.3, and so on).

### Quantitative Analysis of Chaotic Motifs

In the above section, we explored chaotic motifs and explained heuristically the mechanism and structure underlying the characteristics of chaotic motifs. In this section, some quantitative analysis and description are made on chaotic GRNs. The basic idea is that: since chaos is due to the complicated competitions among various oscillatory modes and the intricate drivings among different nodes, it is crucial to explore the driving relationships in GRNs quantitatively. Therefore, we apply the concept of dominant phase-advanced driving (DPAD) [34], [35] and extend the method of DPAD to DPAD time fraction (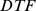). DPAD is a dynamical method that can find the strongest cross driving of the target node at any time instant when a system is in an oscillatory state, and 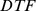 (ranged from 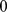 to 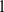) describes the driving contributions of all cross interactions to the target node during some given long period. The max value of 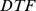 is one, which corresponds to there is only a single cross interaction to the target node; if the value is nearly zero, it means the driving effect of the given interaction to the target node is very weak. For the detail of these concepts, please refer to DPAD and 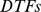 in Materials and Methods.

### Chaotic Motifs Described by 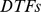


**Figure 5:**
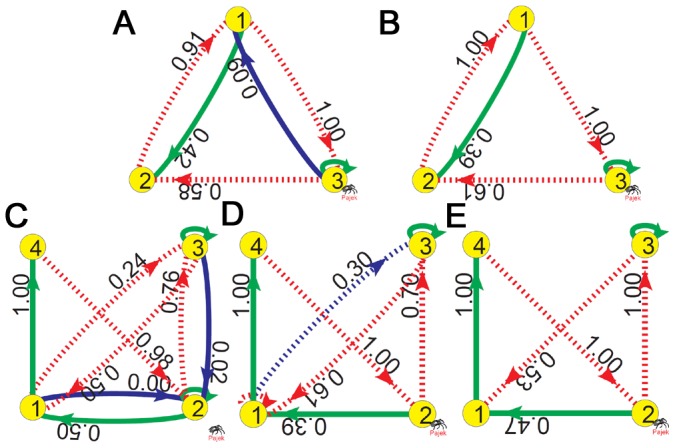
Chaotic motifs described by 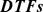. 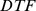
 distributions of the chaotic GRNs of Figs.1 and 2. A for Fig.1A; B for Fig.1D; C for Fig.2A; D for Fig.2B; E for Fig.2C. The numbers associated to all cross interactions indicate the 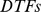 of Eq.(7). The total period of measurement is about 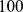 cycles of chaotic orbits. It is shown that most of the interactions reducible for chaos have almost zero 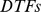, while all the interactions irreducible for chaos in the chaotic motifs in Fig.1D and Fig.2C have sufficiently large 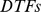. Note that, the two interactions 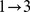 and 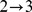 in D have comparable 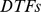. Discarding different one of them can construct different chaotic motifs (motifs (22) and (67) in Fig. S1 by discarding 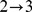 and 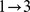, respectively). On the other hand, both the interactions of 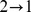 and 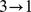 in D are important for the competition between the two loops and thus essential for chaos. There is only one loop in the GRN after removal of 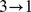 (breaking condition (i)); and the PFL is included in the NFL after deletion of 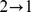 (breaking condition (iii)), and both of the two operations can securely suppress chaotic motions.

It is remarkable that all the chaotic motifs discovered in Figs.1, 2 and 3 can be well explained by the driving relationships where the cross interactions are quantitatively described by 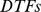. We reproduce the chaotic states of Figs.1C and 1F respectively, and calculate the 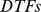 for all cross interactions which are depicted in Figs.5A and 5B. Two interesting features are observed: the interaction 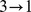 (blue line in Fig.5A) has nearly zero 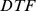 and the other cross interactions of Fig.5A (i.e., all the interactions in the chaotic motif of Fig.5B) have nearly 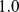 or comparable 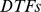. The comparable 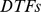 of the interactions 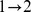 and 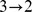 in Fig.5B represent the competition between the two NFLs of 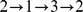 and 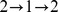 in the chaotic motif of Fig.5B, the key reason for chaos generation. The observations of Figs.5A and 5B quantitatively explain why the interaction 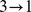 is not crucial for the chaotic behaviors of Figs.1B and 1C and why all the interactions in the chaotic motif of Fig.1D are irreducible for chaotic motions.

In Figs.5C−5E, we do exactly the same as Figs.5A and 5B with the chaotic states of Figs.2G−2I considered. The deleted interactions from Fig.5C to Fig.5D (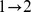 and 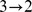, blue lines in Fig.5C) possess near zero 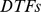, therefore, they have little effect on the chaotic motion of Fig.2G. Note that, the two interactions 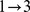 and 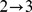 in Fig.5D have comparable 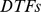. The chaotic motif of Fig.5E (i.e., motif (67) in Fig. S1) is obtained by removing the interaction 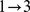, while another motif (motif (22) in Fig. S1) can be also found after removal of 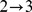. Conversely, the interactions of 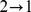 and 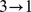 in Fig.5D are both important for chaos (for yielding competition between two loops), and discarding any of them can definitely destroy competition and thus suppress chaos. The reasons are: there is only one loop in the GRN after removal of 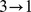 (breaking condition (i)), and all the nodes of the PFL are included in the NFL after deleting 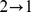 (breaking condition (iii)). Chaotic motions can be thus definitely destroyed in both cases.

### Differences of 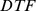 Distributions between Chaotic Motions and Limit Cycles

**Figure 6:**
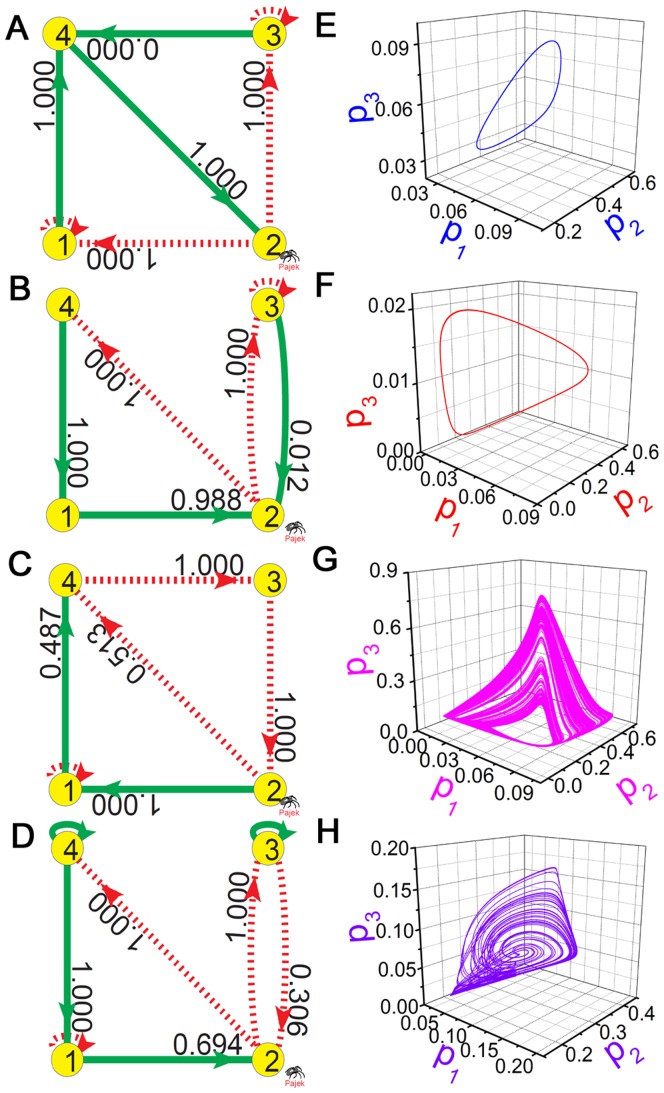
Different 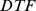 distributions in chaos and limit cycles. A ,B: Two periodically oscillatory TLSs. C ,D: Two 4-node chaotic motifs. E, F: limit cycle solutions of A and B. E with 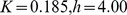 for A; F with 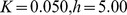 for B. G, H: Chaotic solutions of C and D. G with 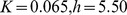 for C; H with 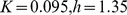 for D. 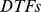 of the corresponding states are labeled on all the cross interactions of the GRNs A-D. It is demonstrated that, there are single effective loops (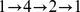 in A and 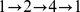 in B) which dominate the oscillation of limit cycles while the two feedback loops possess comparable 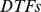 to common nodes (node 4 in C and node 2 in D) in chaotic states.

Besides, it is also interesting to study some distinct differences between simple periodic oscillations and chaotic motions by applying the method of 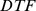. We have a large amount data of limit cycle solutions and chaotic states in TLSs. In Figs.6A and 6B, we show two 4-node TLSs exhibiting simple periodic oscillations in Figs.6E and 6F. The 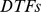 of the two states are depicted in Figs.6A and 6B. Obviously, the interactions with large 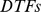 form single loops (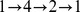 in Fig.6A and 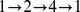 in Fig.6B) and dominate other nodes and the whole networks. Since single strong oscillatory circuit can never produce chaos, the 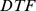 structures of Figs.6A and 6B well explain the periodic behaviors of Figs.6E and 6F. In contrast, the other two 4-node TLSs of Figs.6C and 6D show chaotic motions in Figs.6G and 6H. In Figs.6C and 6D, it is observed that there exist two effective loops with comparative 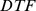 intensities and some common nodes (node 4 in Fig.6C and node 2 in Fig.6D) are driven by two competitive cross interactions (one positive and the other negative). These competitions lead to chaotic motions in Figs.6G and 6H.

### Statistics of Chaotic Motifs in Chaotic GRNs

In the above discussion, chaotic motifs are defined as the minimal and irreducible building blocks for chaotic motions in GRNs. By analyzing the structures of the chaotic GRNs in Table 2, we discover that all the chaotic GRNs have at least one chaotic motif listed in Table 3 and often coupled by multiple chaotic motifs, which together determine dynamics of the networks. Therefore, in the perspective of functional dynamics, a chaotic motif is the necessary condition for chaotic GRNs. Then, we suspect that some optimal motifs may cause chaos with atypically high probability. To confirm this conjecture, we do the following statistical analysis.

For any given GRN, we can find all the TLSs embedded in it. An example of such computation is shown in Fig.8 in Materials and Methods. Accordingly, for a given set of many GRNs, we can sum up the frequency of each embedded TLS, and can compute the relative frequencies of all the 3-node and 4-node TLSs, defined by.
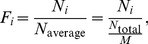
(1)where 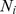 represents the frequency of the 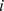
th TLS appearing in the given set of GRNs, 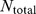 is the total frequency of all TLSs in the set (for 3-node case, 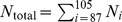, 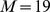; and for 4-node case, 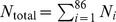, 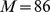). It should be noted that the 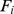 is 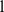 for each TLS in randomly constructed 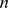-node GRNs with *n*≥4.

**Figure 7:**
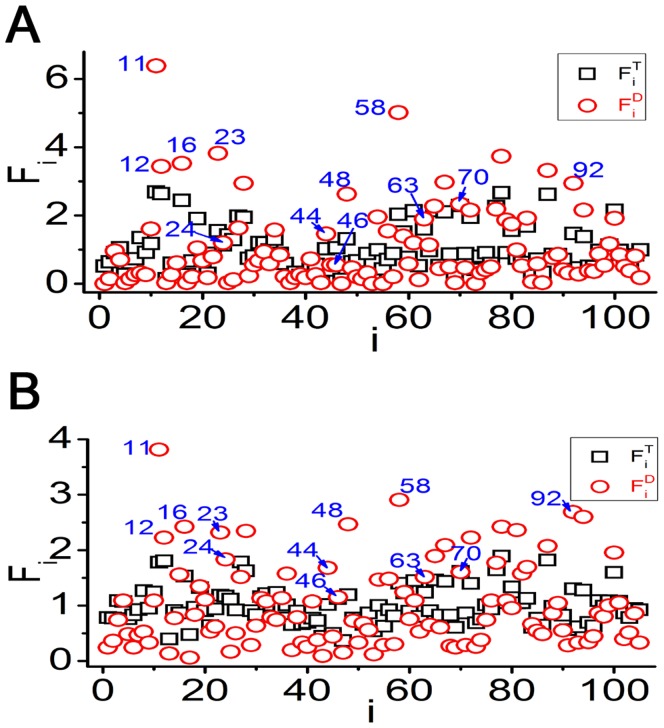
Statistic analysis of chaotic GRNs. A, B: Relative frequency distributions (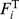
and 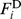) of the 105 TLSs embedded in all chaotic GRNs observed in Table 2 (A for 4-node chaotic GRNs and B for 5-node ones). Black squares represent 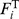 and red cycles denote 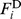, which are computed by Eq.(1) in original topological chaotic GRNs (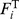) and in dynamically reduced chaotic GRNs by deleting all interactions with 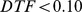 (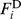), respectively. The significant chaotic motifs in Fig.3 are labeled out with corresponding indexes. Most of them have atypically high 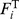 and 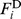, and they more often contain two NFLs. Existence of two independent and competitive oscillatory modes guarantees strong competitions in these subnetworks, leading to chaotic motions.

By the above definition, we can get different 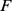 statistics for all TLSs by selecting different sets of GRNs. First, we choose all the chaotic samples of 4-node and 5-node GRNs in Table 2 as given sets, and get the relative frequencies under the original topologies of the chaotic samples, which is denoted by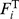. Then, we define dynamically reduced networks, which are obtained by deleting all the unimportant interactions with 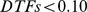 in original topologies of the chaotic samples. The corresponding relative frequencies are defined as 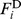. The results of 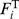 and 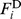 of chaotic GRNs discovered in Table 2 are shown in Figs.7A (4-node chaotic GRNs) and 7B (5-node chaotic GRNs), where black squares denote 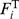 while red cycles 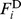. 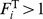 represents that the 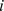
th TLS appears in chaotic GRNs more frequently than the average, and the larger 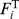 is, the more significantly relevant to chaotic dynamics the 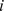
th TLS is. The results of 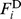 play more important role in characterizing the relevance of given TLSs with chaotic dynamics.

There are usually many feedback loops (far more than two loops) in chaotic GRNs, and the competitions among these loops to produce chaos turn out to be very complicated. The significant motifs effectively producing chaos (shown in Fig.3) are remarked in Fig.7. It is shown clearly that most of the motifs have significant 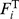 and 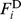. These observations indicate that these significant motifs can most frequently appear in chaotic GRNs and function as the driving sources of chaos. Besides, most of these motifs contain two NFLs. Motifs with two NFLs may easily produce at least two self-sustainedly oscillatory modes in their networks, therefore, the competition between them may easily yield chaos.

It is remarkable that among all the TLSs with 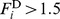 in Figs.7A and 7B, 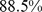 (84.6%) of them are chaotic motifs in Table 3. While among the ones with 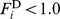, 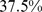 (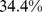) of them are chaotic motifs. All these show the importance of the chaotic motifs in Table 3 to chaotic motions in GRNs.

**Figure 8:**
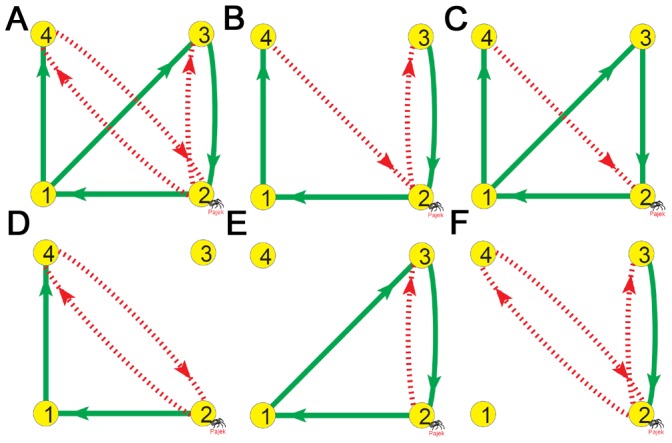
A detailed demonstration of statistical method counting the frequencies of TLSs. A randomly constructed GRN A is considered as an example to show how to compute all the TLSs contained in a GRN. Obviously, there are two 4-node TLSs (B and C) and three 3-node TLSs (D−F) embedded in A. All the 105 TLSs in Fig. S1 are taken into account in our counting. Similar analysis can be applied to all chaotic samples in Table 2 to obtain the results of Figs.7A, 7B.

## Discussion

In conclusion, we studied chaotic behaviors in genomic regulatory systems, and found rich chaotic states in few-node GRNs and TLSs. The rareness of chaos in GRNs is due to the fact that chaos can appear only through competitions among different oscillatory modes under a number of strict conditions. Usually, GRNs do not construct effective oscillatory feedback loops, or build only single strong feedback loops dominating their dynamics [34], [37], resulting in stable steady states and periodic oscillations, respectively. Therefore, no chaos can be generated in these situations. For generating chaos, competitions among different oscillatory modes must fulfill some strict conditions on topological structures, parameter sets and initial conditions. With the clues of these conditions, chaotic behaviors can be easily found in 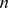
D GRNs with 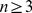.

Chaotic motifs are explored as the minimal and irreducible building blocks satisfying some conditions for chaotic motions (three necessary conditions (i)−(iii) and sufficient conditions (i)−(iv) on the structures of cross interactions) and serve as the intrinsic source of chaos of random few-node GRNs. Some motifs show atypically high probabilities of chaos. Methods of DPAD and 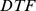 are proposed to explain quantitatively the effects of chaotic motifs. Moreover, we discover that a number of special structures can never produce chaos, and this conclusion may be also important for biological understanding and designment.

Chaotic GRNs usually have very complex structures and contain various regulatory circuits much more than TLSs. It is our future work to recognize the few dominant oscillatory modes essentially determining the chaotic motions from probably large numbers of topological feedback loops in GRNs. DPAD and 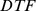 may be very helpful in this research. We discover also that chaotic GRNs are often coupled by multiple chaotic motifs which together determine rich variety of chaotic attractors. Some attractors belong to phase coherent oscillators which have the property of Uniform Phase evolution and Chaotic Amplitudes (UPCA), while some others are not in strict phase-locking (funnel attractor). Some other spiral and screw chaotic attractors are also found. The chaotic attractors we find are nonhyperbolic. Most of the roads to chaos in GRNs are through period-doubling to chaos; intermittency to chaos; and quasi-period to chaos. It will be another task of our research to pursue the topological origins of different classes of chaotic attractors and roads to chaos. We expect to attack this target by applying the methods of DPAD and 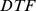.

We find a large set of chaotic TLSs (chaotic motifs) and the topological conditions for chaos in GRNs by simulations. It is interesting whether these indeed make sense in real GRNs. For example, the P53 system, a crucial complex GRNs for regulating cell cycle and suppressing tumor, has many feedback loops. The core regulative structure of P53 system has been pointed out by a recent study [38], it is a four-gene network, containing three NFLs and two chaotic motifs we defined. These indicate that the P53 system could produce chaotic behaviors and is sensitive to initial conditions. Actually, P53 protein (the tumor suppressor) is not robust enough to prevent cancer and conserve stability. Although chaos has never been reported by any genetic experiment, we speculate this may be due to three factors. One may be that the competitions between oscillatory modes is not strong enough. Another may be that chaotic behaviors can appear but can not be sustained for a long time, because the beginning of chaos could induce the activation of other external regulatory pathways (such as the cell-to-cell communication [39]). Some researchers consider that the appearance of chaos is an indication of a diseased state, and is most often connected with the beginning of cancer [40]. The last may be that biochemical noise appears often in living cells, and if the noise on gene expression is strong enough, then it would destroy the chaotic state of a noise-free genetic system. By simply considering Gaussian white noise on above motifs, we find that all the oscillatory and chaotic phenomena observed in the paper are robust against small noise. However, relatively large noise may induce some interesting and unexpected results. Increasing noise may totally suppress chaos and result in noisy steady state. In particular, the effects of same noise on different nodes can be different, depending on the network structures or something else (still not fully understood). Moreover, limit cycles are much more robust against noise than chaotic attractors. However, we still can not declare that there is no chaos in GRN, because it is not easy to evaluate the strength of noise. In the next step, we will systemically investigate the effects of noises on dynamical behaviors of GRNs, and combine some experimental data to find whether chaotic motifs have realistic effects on the function of living systems.

We hope that the findings in the present work may give some impacts on the investigation of extremely rich behaviors of nonlinear dynamics and pattern formation in various biochemical systems, and contribute to exploring mystery biological functions and effects caused by chaotic regulatory networks.

## Materials and Methods

### Model

In this paper, we discuss the chaotic dynamics of few-node autonomous GRNs by using a model of ODEs. The main conclusions of this article do not depend on the detail of the model, and can be extended to a class of models with monotonic regulatory functions. We use the following simplified genomic regulatory model [12], [13], [34], [41], [42].
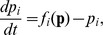


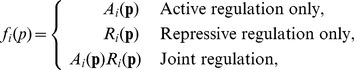


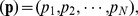


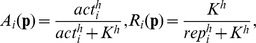


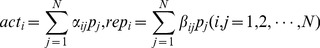
(2)where 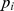 is the expression level of gene 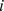, 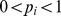. The adjacency matrix 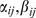 determine the network structure of the system, which are defined in such a way that 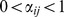 if gene *j* activates gene *i*, 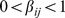 if gene 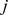 inhibits gene 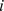 and 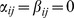 for no regulation of gene 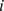 by gene 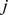. For convenience, all the corresponding 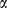 or 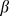 in Figs.1 and 2 are set to 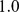. 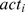 (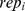) represents the sum of active (repressive) transcriptional factors to node 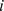. The regulatory interactions of genes are represented by Hill functions with the cooperativity exponent 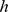 (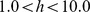) and the activation coefficient 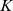 (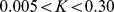), characterizing many real genetic systems. The model is of no-delay monotonous regulation. We neglect the leakage transcription rate, and the degradation rates of different proteins are identical.

Searching for chaos: (i) In Table 1, network structures are randomly generated but to make sure that each node in the network has at least one input and at least one output (all the nodes are linked together). All the parameters 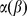, 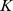 and 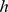 are randomly given within their ranges. (ii) In Table 2, to enlarge the chaotic samples, oscillatory network structures in Table 1 are used to search for chaos. 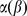 of corresponding networks are remained, 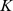 and 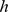 are randomly given. (iii) In Table 3, chaotic samples are searched among the 105 TLSs with 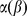, 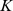 and 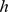 given randomly.

### DPAD and *DTFs*

The idea to make the DPAD analysis is that any single node of a GRN (node 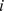 for example) can not oscillate individually, and it can oscillate only through the driving of cross interactions from other nodes in the network. At any time instant (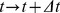), node 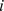 undergoes the cross driving 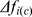,
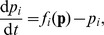


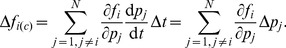
(3)


In the total cross driving signal of node 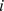, the fraction of the contribution from node 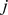 over 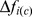 can be computed.
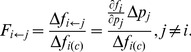
(4)


All the cross interactions to node 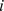 (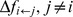) can be classified into two distinct types: one is favorable to the cross driving (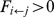) while the other not (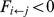). We can define the former as “phase-advanced drivings” (PADs) while the latter “phase-delayed drivings”. A node may be driven by a number of PADs among which a single PAD can be found providing the largest 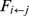 value to 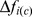. This most important PAD is called DPAD which can be calculated at any time instant. We can quantitatively compute the DPAD contribution of any node 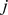 to the target node 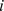 at a time step 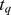,
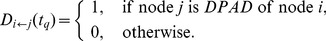
(5)


Now, we define a DPAD weighted time interval of node 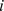 as.
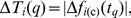
(6)with 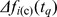 being taken at the time interval 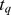. Then, we can further measure DPAD time fraction (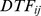) for a given interaction from node 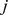 to play the role of DPAD on the driven node 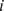 over certain long time (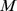 time steps, 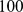 cycles in our measurement)



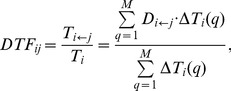
(7)

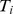
 is the total measuring weighted time of node 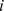, while 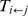 represents the sum of weighted time interval in 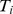 when the interaction from node 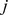 to node 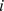 plays the role of DPAD (i.e., all 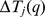 when 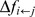 is DPAD). 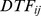 measures the contribution of node 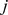 in driving node 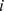 to oscillation quantitatively.

### The Detailed Course of the Statistical Method

A randomly constructed GRN of Fig.8A is considered as an example to show how to compute all the TLSs a GRN contains. Obviously, there are two 4-node TLSs (Figs.8B and 8C) and three 3-node TLSs (Figs.8D−8F) embedded in the GRN of Fig.8A. All the 105 TLSs in Fig. S1 are taken into account in our counting. Similar analysis can be also made on a large number of GRNs, and the relative frequencies in Eq.(1) of the 105 TLSs appearing in these GRNs can be computed.

### Supporting Information

#### The 105 TLSs

All the chaotic motifs may possess the following two characteristic features: (i) They have two feedback loops (the fewest feedback loops); (ii) At least one of the two loops is NFL. We exhaustively search all 3-node and 4-node two-loop structures satisfying the above two conditions. Fig. S1 shows the complete set of all these 105 distinctive two-loop structures (TLSs).

## Supporting Information

Figure S1
**The 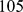 two-loop structures (TLSs).** Complete set of distinctive 3-node and 4-node TLSs satisfying conditions (i) and (ii), serve as possible candidates of chaotic motifs. All the 105 TLSs are listed (4-node (1)−(86) and 3-node (87)−(105)) with the corresponding indexes used in the text and other figures.(EPS)
